# Diagnostic accuracy of presepsin for sepsis in adult patients according to the criteria of sepsis-3: systematic review and meta-analysis

**DOI:** 10.1186/s12871-026-03983-w

**Published:** 2026-06-19

**Authors:** Sedigheh Damavandi, Hassan Emami, Farkhondeh Asadi, Elham Nazari, Shahabedin Rahmatizadeh

**Affiliations:** Department of Health Information Technology and Management, School of Allied Medical Sciences, Shahid Beheshti University of Medical Sciences (SBMU), Tehran, Iran

**Keywords:** Presepsin, Sepsis-3, Diagnostic accuracy, Meta-analysis

## Abstract

**Background:**

Time-sensitive identification of sepsis plays an essential role in minimizing sepsis-related death. One of the latest and important markers recognized is presepsin, also known as sCD14-ST. After the publication of the Sepsis-3 criteria in 2016, there have been discrepancies found among meta-analyses using both old and new criteria combined. The purpose of this current analysis is to evaluate the performance of presepsin at presentation, using a scientific approach and only considering studies conducted using Sepsis-3 criteria.

**Methods:**

Electronic searches of PubMed, Scopus, Embase, Web of Science, and the Cochrane Library were carried out until 10 December 2025. Prospective and retrospective studies that expressed the sensitivity and specificity of plasma presepsin for sepsis diagnosis in adults (aged ≥ 18 years) based on Sepsis-3 criteria were considered for review. Bivariate random-effects model estimates of sensitivity, specificity, DOR, and SROC plot were calculated.

**Results:**

Eleven studies (2248 participants) were included. Pooled sensitivity was 0.782 (95% CI: 0.755–0.807), and pooled specificity was 0.739 (95% CI: 0.714–0.763). The area under the SROC curve was 0.85 (good diagnostic accuracy). The negative likelihood ratio was 0.297 (95% CI: 0.240–0.367), indicating a useful rule out test. Heterogeneity was substantial (I² > 75% for sensitivity and specificity). There was no evidence of a threshold effect based on Spearman correlation (*p* = 0.326), although Moses’ model suggested a mild threshold effect (*p* = 0.018). Meta regression showed that the cut off value did not significantly influence diagnostic accuracy (*p* = 0.878).

**Conclusions:**

Presepsin has a good diagnostic accuracy for sepsis in adults and is a useful rule-out test. Presepsin has good diagnostic accuracy for sepsis in adults and is a useful rule out test. However, wide variation in cut off values across studies underscores the need for standardization before widespread clinical adoption.

## Intro

Sepsis may lead to organ failure and ultimately result in death. It is one of the main reasons for high morbidity rates among the community of such patients across the world. It has been argued that the estimated pool is roughly 49 million people who are affected every year, resulting in a death toll of 11 million people according to the World Health Organization and the Global Burden of Disease on the subject of Sepsis. Thus, according to global figures, it is estimated that a considerable 20% of global deaths occur. Such countries with lower and middle economies have a greater burden as far as resource availability for the purpose of quicker diagnosis and treatment is concerned [1]. However, high mortality rates remain despite advancements in intensive care. Generally speaking, in severe cases, the mortality rate is greater than 20–30%, which increases further for patients suffering from septic shock at a rate of 40–50%. It translates the importance of this fast yet accurate method for the purpose of effective immediate action [2, 3]. Sepsis is considered to be the most difficult diagnosis, as symptoms occur that are nonspecific or those of non-infectious systemic inflammatory response syndrome (SIRS). Further, the standard blood analysis is time-consuming as well. It typically takes anywhere between 24 and 72 h. Rather often, it turns out to be false negatives [2, 4]. By the year 2016, early monitoring biomarker it was the “Third Annual General Consensus” referred to as “Sepsis-3” which brought a new “paradigm” as far as the subject is concerned: that is, taking cognizance of the concept of “organ failure” rather than “inflammation.” Thus, this would involve the suggestion regarding the “use” of “Sequential Organ Failure Assessment” scores and other techniques. Thus, this would resolve at least some of the greatest problematic aspects of the “Sepsis-1” and “Sepsis-2” definition, which relied upon SIRS. For the treatment of Sepsis, “biomarkers” emerged as vital agents for earlier diagnosis and treatment [2, 5, 6]. Whereas present biomarkers such as C-Reactive Protein (CRP) [7] and procalcitonin levels are already in extensive use by specialists across the world, their advantages remain somewhat restricted for reasons such as “moderately low specificity” or “kinetics” [8–10].

Recently, the interest has focused on the novel, promising biomarker, namely presepsin or sCD14-ST [11]. It is created as a consequence of CD14 receptor fragmentation, where an elevation in levels occurs early on, that is, within 2–3 h after entering the body, thus triggering the innate immune response due to the entry of microbes [4]. Some promising outcomes have been observed in the earlier meta-analysis regarding its accuracy for presepsin, but the outcome got constrained due to extreme unpredictability in higher heterogeneity due to the allocation regarding incongruent or outdated sepsis classification categories, that is, Sepsis-1/2 or Sepsis-3 among them in these studies [10, 12]. Consequently, due to the consolidation of different criteria, it became difficult to interpret them. The most recent among these, that is, the Bayesian network meta-analysis in 2025, assessed and supported the strength regarding the diagnosis of presepsin [13], but none existed regarding studies strictly within the boundaries of the Sepsis-3 criteria among adults.

To fill this gap, this systematic review and meta-analysis will attempt to merge studies that assessed the diagnostic accuracy of presepsin only in adult patients using plasma measurements with the Sepsis-3 definition. The primary goal is to use this meta-analysis to develop reliable estimates for the pooled value of the sensitivity, specificity, positive likelihood ratio, negative likelihood ratio, diagnostic odds ratio (DOR), and area under the SROC. It is believed that, through the study of homogeneous studies and by investigating the components of heterogeneity, the definitive importance of presepsin for the early diagnosis of sepsis among the study population can be elucidated.

## Methods

### Study design & reporting guidelines

This systematic review and meta-analysis were done following the Preferred Reporting Items for Systematic Reviews and Meta-Analyses of Diagnostic Test Accuracy Studies (PRISMA-DTA). Although the review protocol was not registered in PROSPERO (a limitation acknowledged in the Discussion), the search strategy, eligibility criteria, and analysis plan were specified a priori to enhance transparency and reduce the risk of bias.

### Search strategy and study selection

A thorough search of the electronic literature bases PubMed, Scopus, Embase, Web of Science, and the Cochrane Library was conducted from the earliest possible database inception through the cut-off date of 10 December 2025. The search strategy included Medical Subject Headings (MeSH) terms and text words for the index test (“presepsin,” “sCD14-ST”), the targeted condition (“sepsis,” “septic shock”), and diagnostic accuracy (“diagnostic accuracy,” “sensitivity”). Manual searching of the reference lists for relevant reports and abstracts was also done.

### Requirements for eligibility

The PICOS criteria were used to establish which studies were eligible for inclusion in the meta-analysis:

The target population for the research includes all adult patients aged 18 years and above who have sepsis symptoms, which are confirmed by the increase of SOFA score by 2 or more under the Sepsis-3 criteria.

The Index test (I) shows the plasma presepsin concentration values where the thresholds for the diagnosis have been determined by the findings of research work.

The reference standard for the comparator includes patients with sepsis, which was confirmed by the clinicians and microbiological analysis based on the Sepsis-3 criteria.

The research must yield sufficient data, which will allow the researchers to construct a 2 × 2 contingency table for data analysis or a measure for the degree of accuracy of the diagnosis—true positives, false positives, false negatives, and true negatives.

The research design includes observational studies, which may work on either prospect or a retrospective design. The analysis does not include studies based on previous sepsis criteria, such as Sepsis-1/2, unless the researchers have offered data specifically for the Sepsis-3 patients. The analysis does not include review articles, case reports, and animal studies, nor research that involved only children.

### Process for study selection

**Fig. 1:**
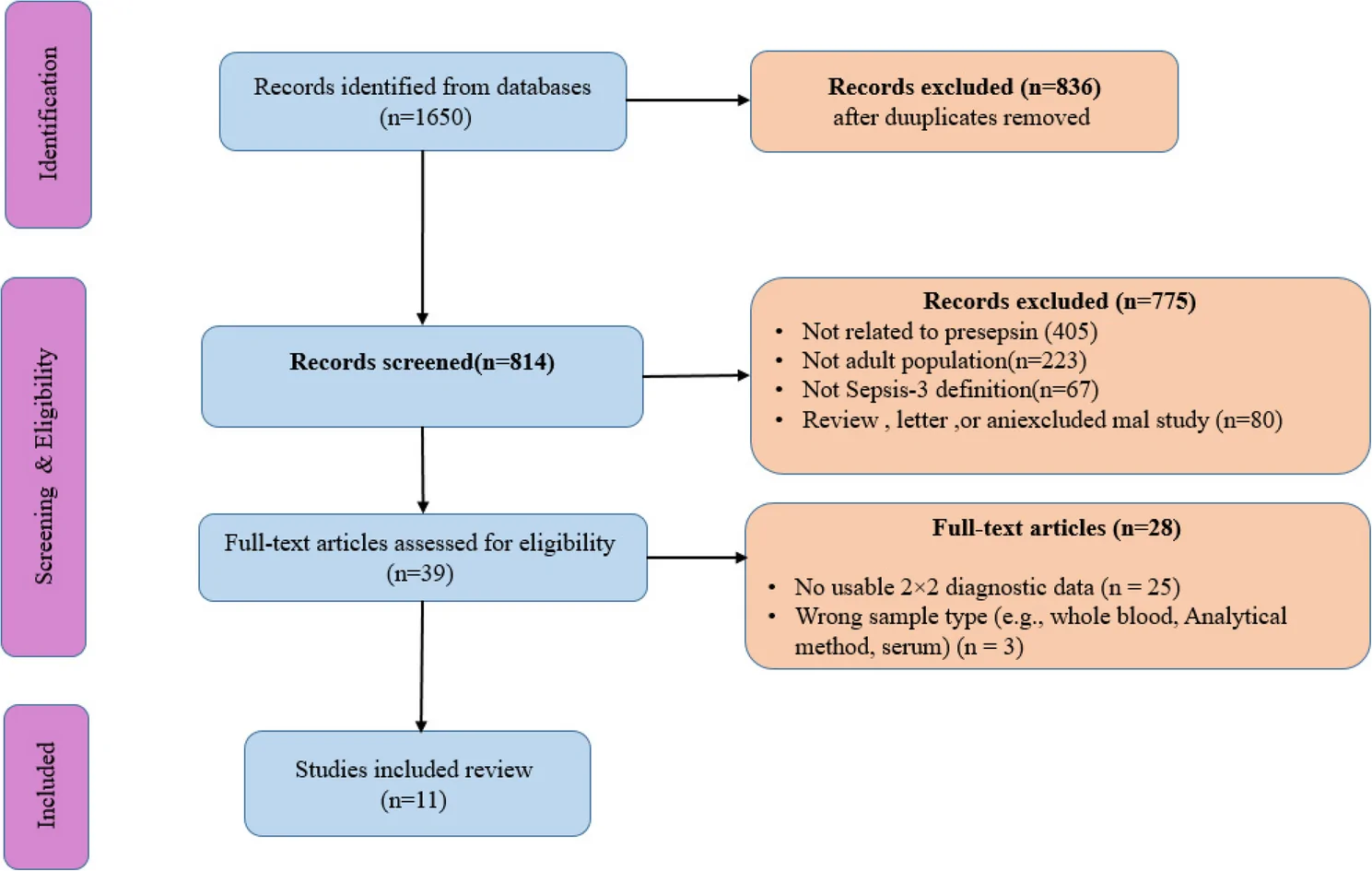
PRISMA flow diagram of the study selection process

S.D and E.N chose the studies they wanted to include for analysis using the Rayyan software. They read the abstracts and titles before deciding to read the full text of studies that were likely to meet the inclusion criteria. They discussed any discrepancies regarding the inclusion of studies and reached a consensus on whether a study should be included until they required the help of a third party. The selection process is summarised in the PRISMA flow diagram (Fig. 1), which has been updated to reflect the final 11 included studies.

### Data extraction

Two authors independently extracted data using a standardized, piloted form developed in Microsoft Excel. The data that were extracted were characteristics of the studies (first author, year, country, type, and clinical setting), characteristics of the participants (sample size, age, and gender), and key data related to diagnostic accuracy. The diagnostic accuracy data were raw data values for constructing 2 by 2 tables (TP, FP, FN, TN), threshold values of presepsin, test type, and evidence that the sample type was plasma. When there was more than one threshold value, the authors’ “optimal” threshold values were selected for the meta-analysis. Other data values, such as those related to AKI, were also extracted but only used for sensitivity analysis [9, 14]. When values were ambiguous, corresponding authors were approached a maximum of two times for further information. When there was no response, the data point was not used in the quantitative synthesis. To prevent bias, values expressed numerically were always preferred over values derived graphically.

### Quality evaluation

**Fig. 2:**
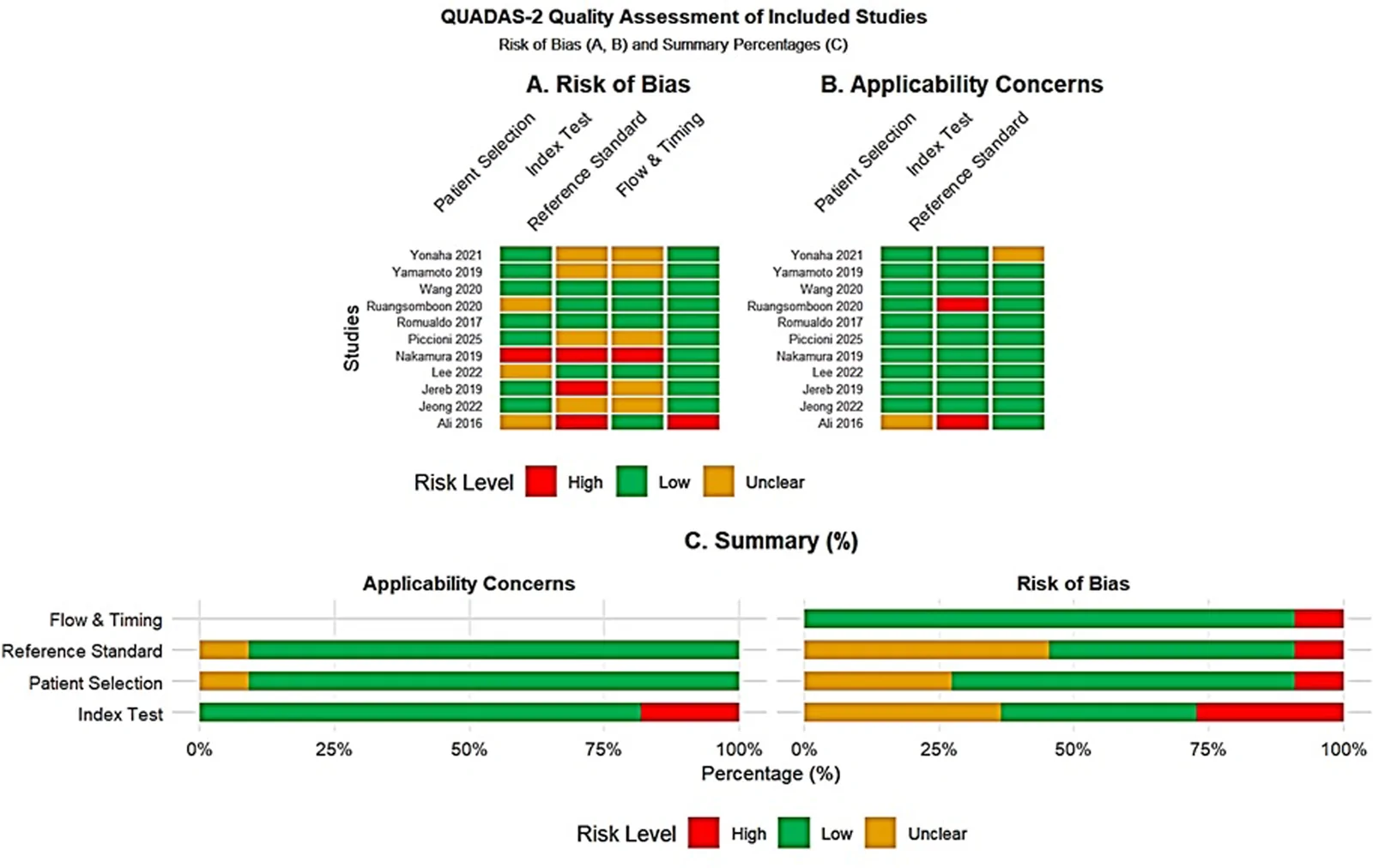
Summary of quality assessment for 11 paper

Two reviewers applied the Quality Assessment of Diagnostic Accuracy Studies-2 (QUADAS-2) to evaluate the methodological quality of the included studies. This instrument considers the risk of bias in four domains: patient selection, index test, reference standard, and flow and timing. In addition, this instrument considers concerns about applicability in three domains. This instrument used the signaling questions to evaluate the risk of bias in each domain as “low,” “high,” or “unclear.” “High” risk of bias was assigned if there was an answer of “no” to at least one key question. “Unclear” risk of bias was assigned if there was insufficient information to make an evaluation. Disagreements between reviewers were resolved in discussions, and if necessary, a third reviewer was consulted (Fig. 2).

### Statistical analysis

The bivariate random-effects model with restricted maximum likelihood (REML) estimation was applied to pool sensitivity, specificity, positive likelihood ratio (PLR), negative likelihood ratio (NLR), and diagnostic odds ratio (DOR). This model accounts for the correlation between sensitivity and specificity and for within- and between-study variability. The hierarchical summary receiver operating characteristic (HSROC) curve was plotted, and the area under the curve (AUC) was calculated. Heterogeneity was assessed using the I² statistic (values > 50% indicating substantial heterogeneity) and τ².

All analyses were performed using Meta-DiSc version 1.4, which implements the bivariate random-effects model for pooled estimates. The Moses-Littenberg model was used only to investigate the presence of a threshold effect (by regressing the logit of the DOR against the logit of sensitivity). Spearman’s correlation between the logit of true-positive rate and logit of false-positive rate was also calculated to assess threshold effects.

Meta-regression was conducted using the covariate Log(Cutoff) to explore whether the presepsin threshold value influenced diagnostic accuracy. For studies reporting multiple cut-offs, the authors’ “optimal” threshold (usually based on the Youden index or clinical judgment) was selected. Zero cells in 2 × 2 tables were corrected by adding 0.5. Publication bias was evaluated using Deeks’ funnel plot asymmetry test (*p* < 0.10 considered significant).

### Ethical considerations

As a secondary analysis of a published dataset, there was no need for formal approval through an institutional review board. This research fully meets the known ethical guidelines for the conduct and reporting of systematic reviews.

## Results

### Study selection and characteristics

After removing duplicates, 814 records were screened. Following title/abstract and full‑text review, 11 observational studies encompassing 2,248 patients (996 with sepsis, 1,252 without) were included (Fig. 1). All studies used the Sepsis‑3 definition. Presepsin was measured by CLEIA (11 studies). Cut‑off values ranged from 165 to 1,315 pg/mL.

### Quality assessment

Two reviewers applied the Quality Assessment of Diagnostic Accuracy Studies-2 (QUADAS-2) to evaluate the methodological quality of the included studies. This instrument considers the risk of bias in four domains: patient selection, index test, reference standard, and flow and timing. In addition, this instrument considers concerns about applicability in three domains. This instrument used the signaling questions to evaluate the risk of bias in each domain as “low,” “high,” or “unclear.” “High” risk of bias was assigned if there was an answer of “no” to at least one key question. “Unclear” risk of bias was assigned if there was insufficient information to make an evaluation. Disagreements between reviewers were resolved in discussions, and if necessary, a third reviewer was consulted (Fig. 2; Table 1).

**Table 1:** Baseline characteristics of the included studies

	Author	Year	Country	Population	Diagnostic criteria	Study design	Data sources	Clinical setting	Specimen type	Analytical method	Case	Control	Cut-off (pg/mL)	Sensitivity	Specificity	AUC	TP	FP	FN	TN
1	Ruangsomboon [15]	2020	Thailand	Adult (≥ 75 y)	Sepsis-3	Prospective	Single-center	Emergency Dept (Non-ICU)	Plasma	CLEIA	139	111	380	0.835	0.622	0.792	116	42	23	69
2	Piccioni [16]	2025	Romania	Adult	Sepsis-3	Prospective	Single-center	Emergency Dept (Non-ICU)	Plasma	CLEIA	122	94	165	0.910	0.202	0.946	111	75	11	19
3	Jeong [17]	2022	Korea	Adult (post-abdominal surgery)	Sepsis-3	Prospective	Single-center	ICU	Plasma	CLEIA	91	169	314	0.698	0.614	0.668	64	65	27	104
4	Jereb [18]	2019	Slovenia	Adult	Sepsis-3	Prospective	Single-center	ICU (Infectious Diseases)	Plasma	CLEIA	54	26	751.5	0.833	0.962	0.832	45	1	9	25
5	Ali [5]	2016	Egypt	Adult	Sepsis-3	Prospective	Single-center	ICU	Plasma	CLEIA	33	18	907	0.697	0.833	0.805	23	3	10	15
6	Lee [19]	2022	Korea	Adult	Sepsis-3	Prospective	Single-center	Emergency Dept	Plasma	CLEIA	278	142	582	0.701	0.894	0.877	195	15	83	127
7	Yonaha [20]	2021	Japan	Adult	Sepsis-3	Prospective	Single-center	ICU	Plasma	CLEIA	56	42	1315	0.893	0.500	0.682	50	21	6	21
8	de Guadiana [21]	2017	Spain	Adult (≥ 14 y)	Sepsis-3	Prospective	Single-center	Emergency Dept (Non-ICU)	Plasma	CLEIA	70	130	849	0.671	0.808	0.775	47	25	23	105
9	Wang [22]	2020	China	Elderly (≥ 65 y, ICU)	Sepsis-3	Prospective	Single-center	ICU	Plasma	CLEIA	44	98	434	0.821	0.897	0.901	36	10	8	88
10	Nakamura [23]	2019	Japan	Adult (ICU, non-AKI subgroup)	Sepsis-3	Retrospective	Single-center	ICU	Plasma	CLEIA	47	393	240	0.809	0.832	0.880	38	66	9	327
11	Yamamoto [24]	2019	Japan	Adult (ICU, SIRS+SOFA ≥ 2)	Sepsis-3	Prospective	Single-center	ICU	Plasma	CLEIA	62	29	508	0.870	0.860	0.880	54	4	8	25

### Pooled diagnosis performance

**Fig. 3:**
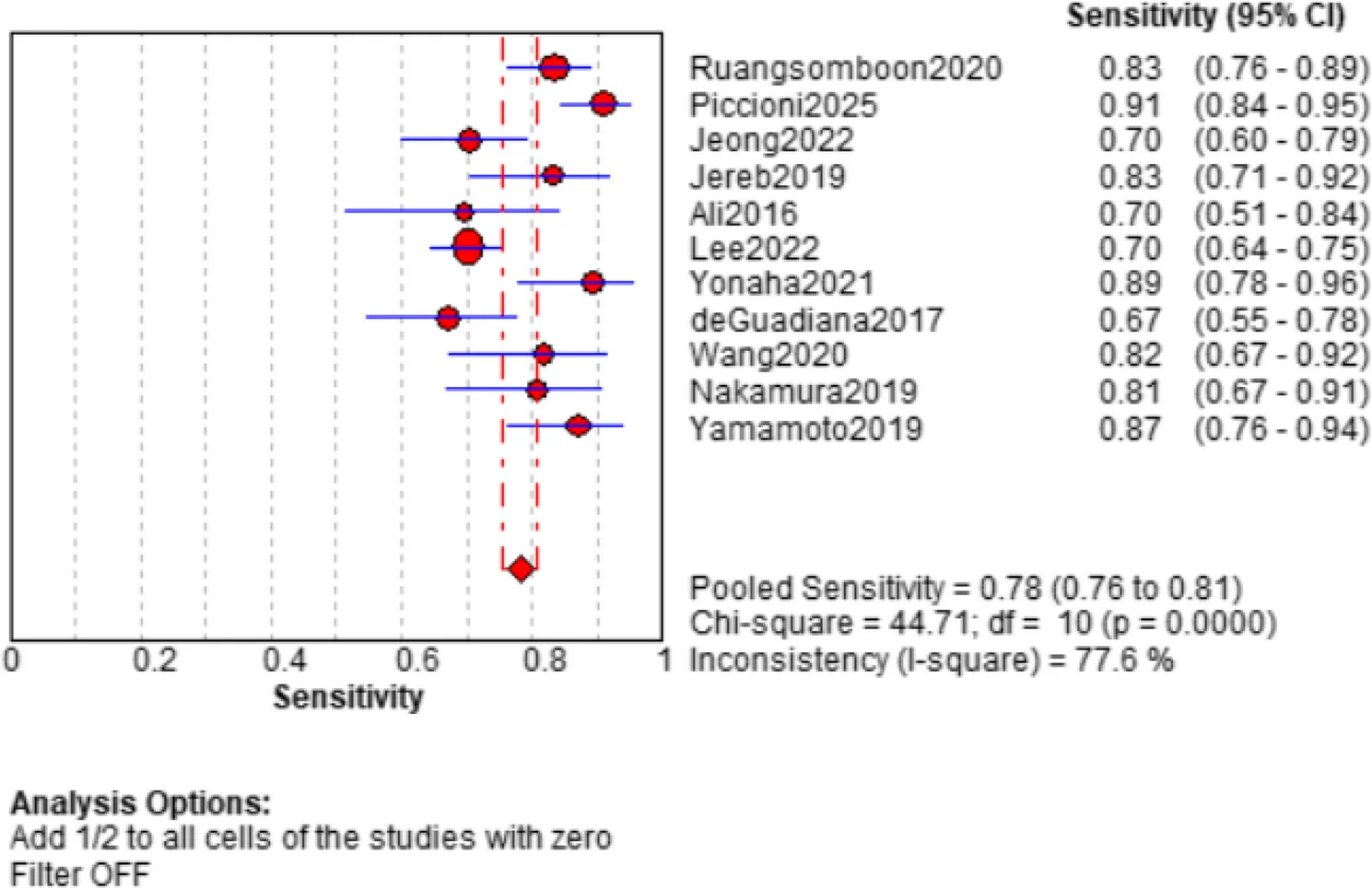
Forest plot of pooled sensitivity

**Fig. 4:**
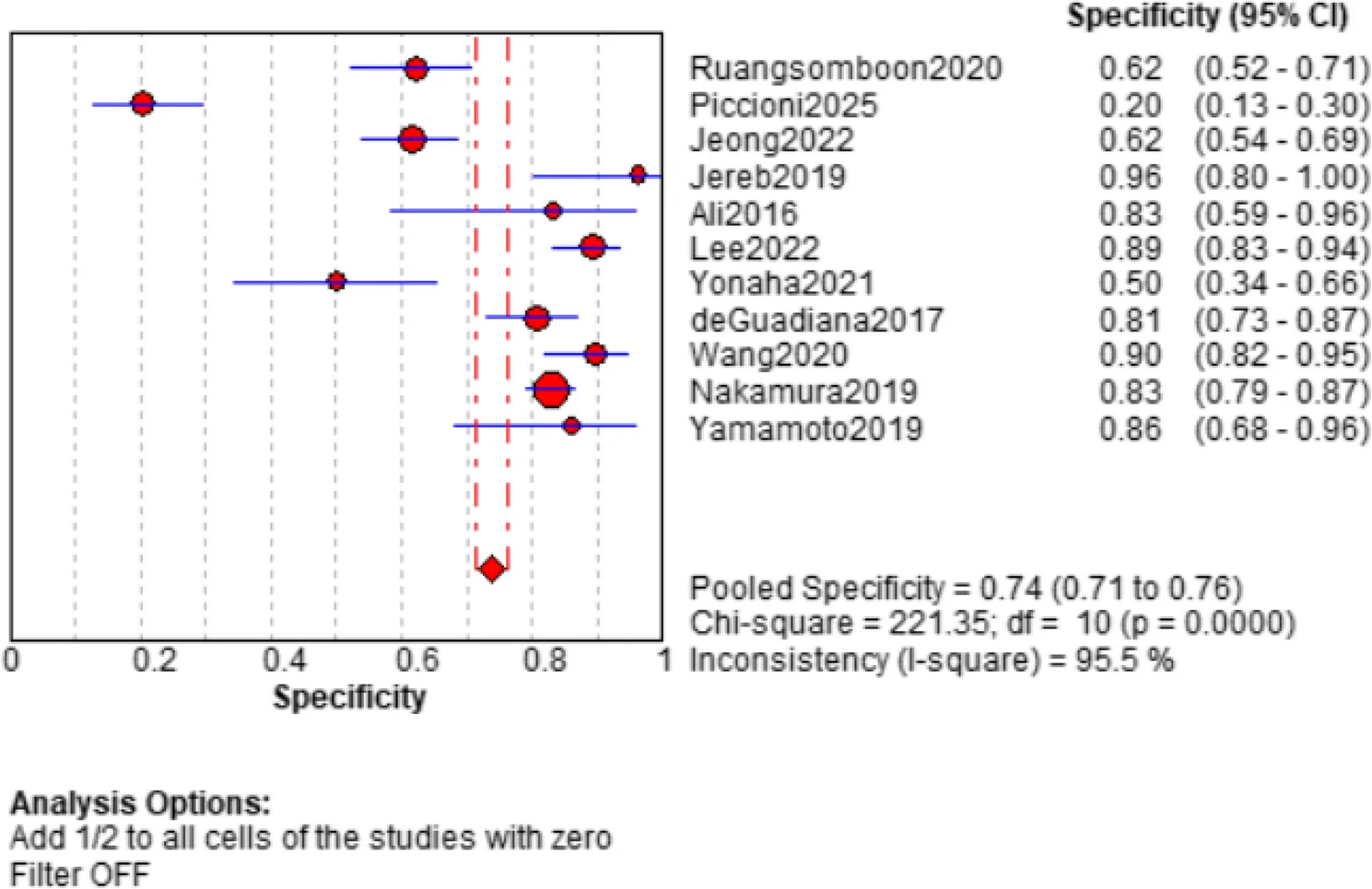
Forest plot of pooled specificity

**Fig. 5:**
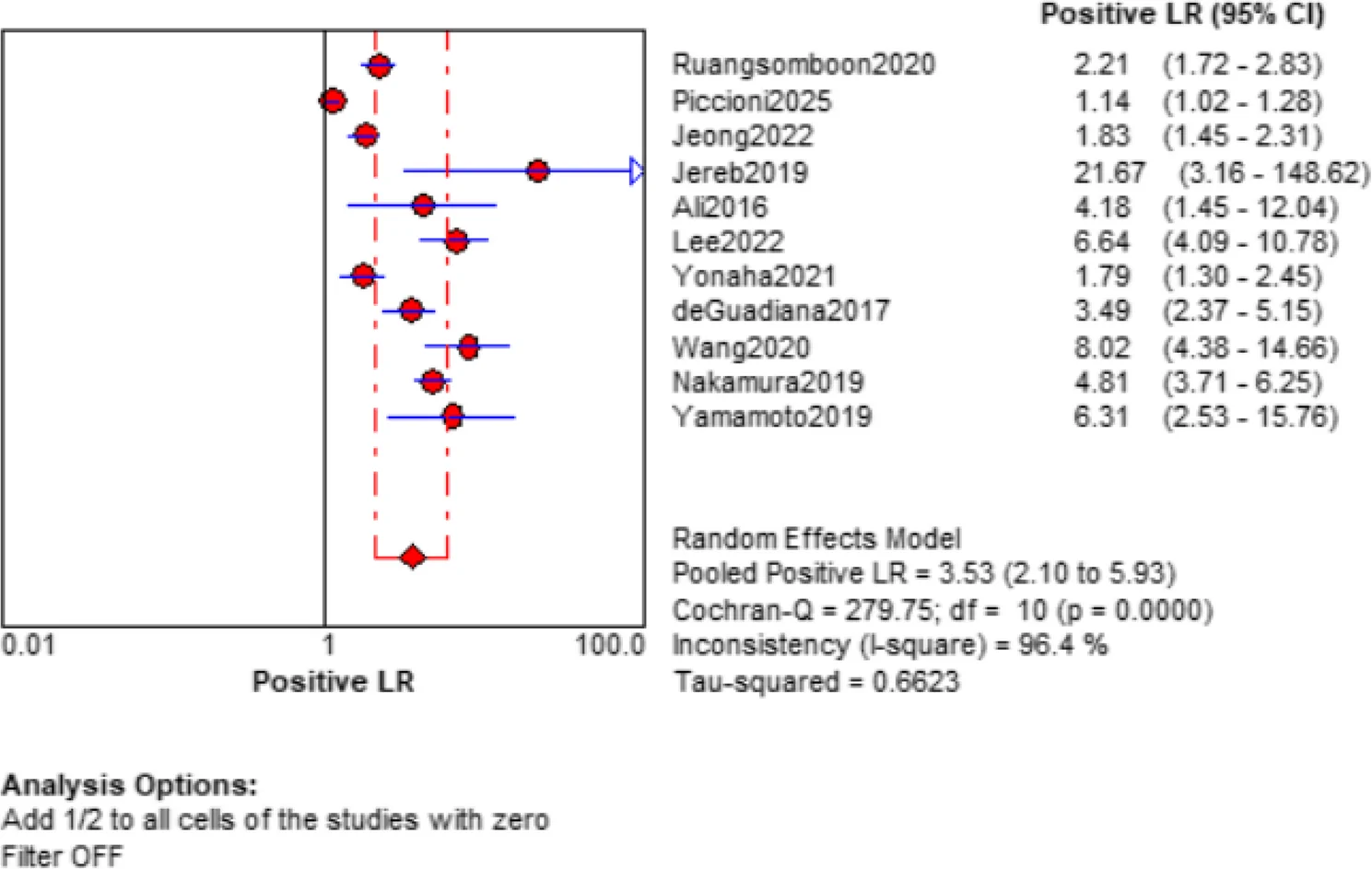
Forest plot of positive likelihood ratio

**Fig. 6:**
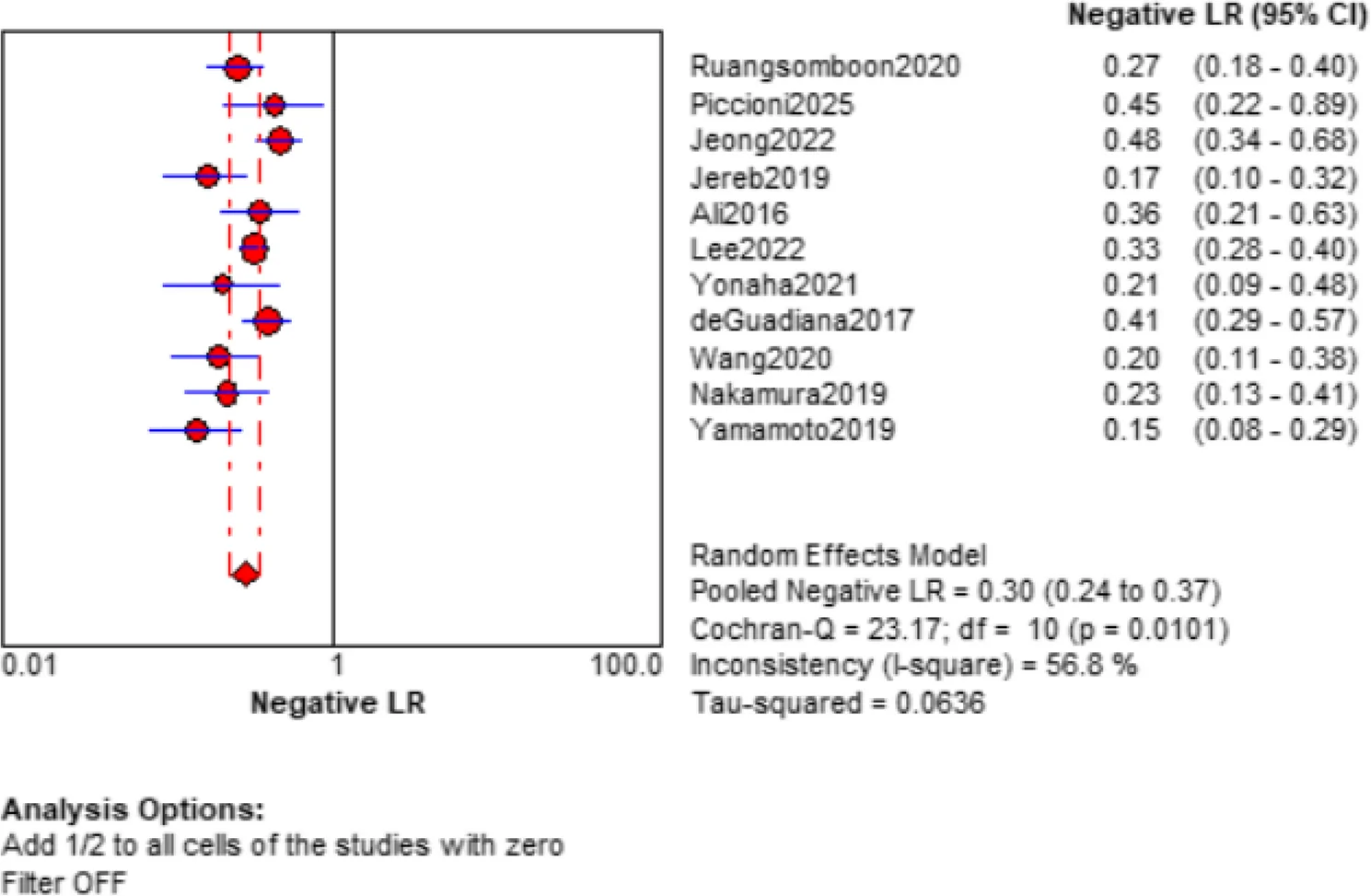
Forest plot of negative likelihood ratio

**Fig. 7:**
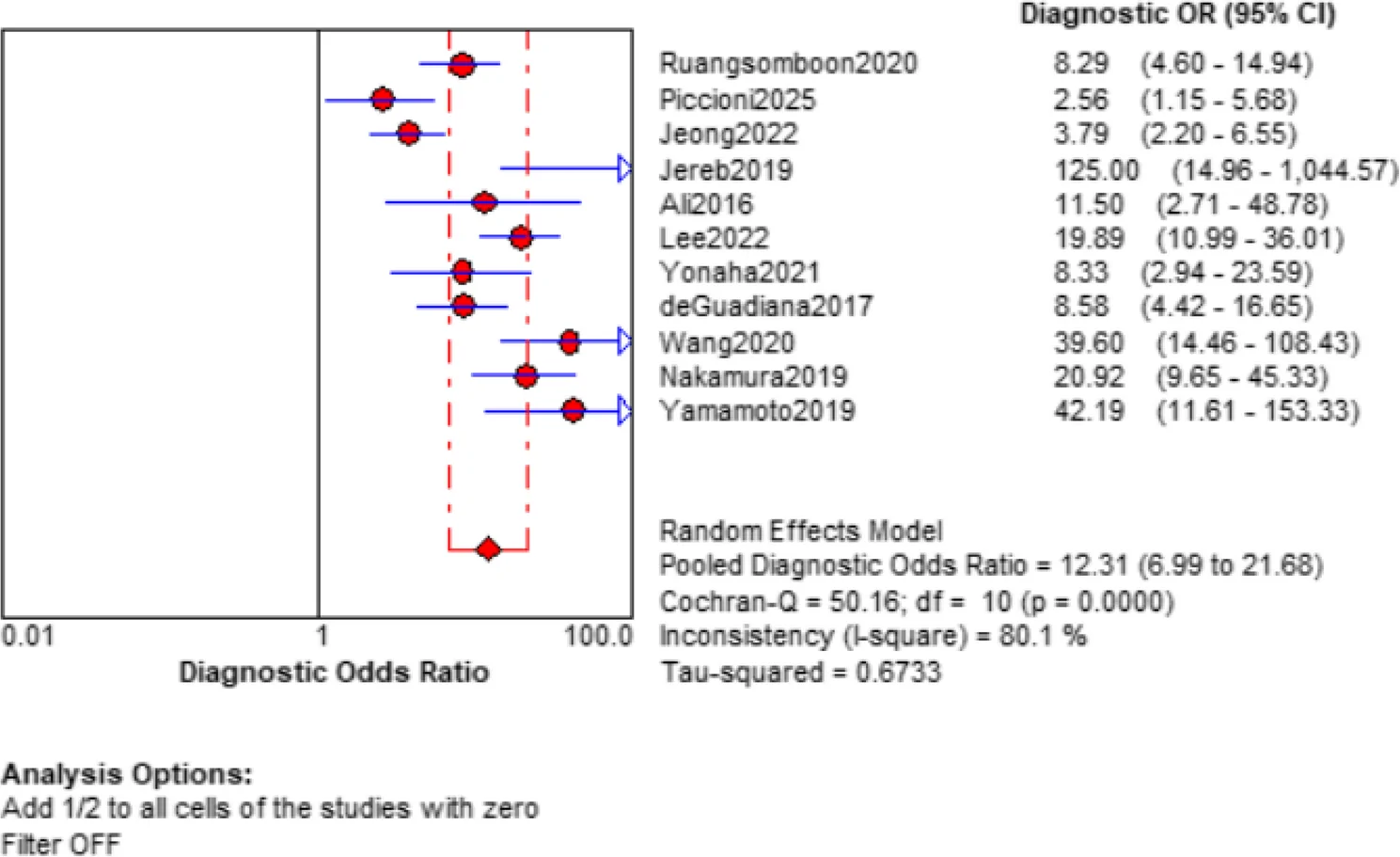
Forest plot of diagnostic odds ratio

**Fig. 8:**
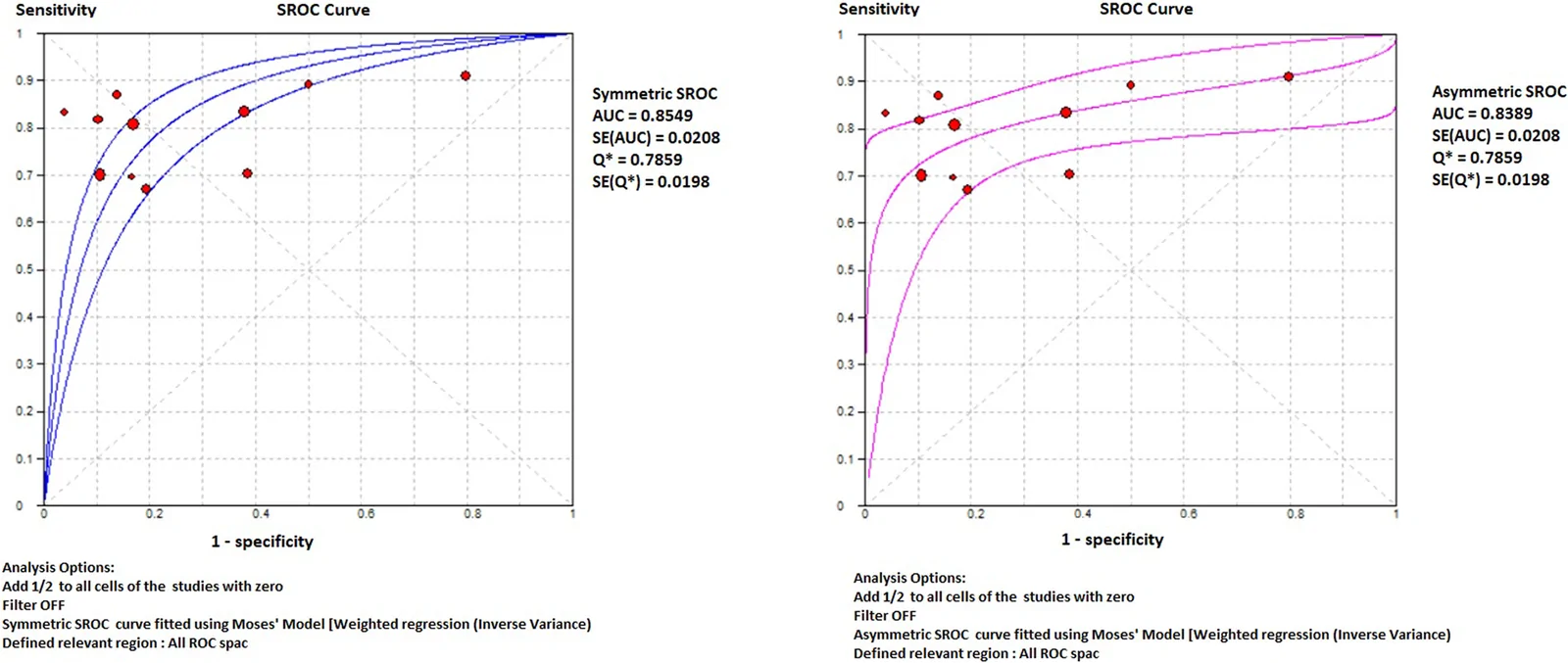
.Figure 8: Summary receiver operating characteristic (SROC) curve

Using the bivariate random-effects model, the pooled sensitivity of presepsin was 0.782 (95% CI: 0.755–0.807) with substantial heterogeneity (I² = 77.6%; Fig. 3). The pooled specificity was 0.739 (95% CI: 0.714–0.763) with considerable heterogeneity (I² = 95.5%; Fig. 4). The positive likelihood ratio (PLR) was 3.53 (95% CI: 2.10–5.93; I² = 96.4%; Fig. 5), and the negative likelihood ratio (NLR) was 0.297 (95% CI: 0.240–0.367; I² = 56.8%; Fig. 6), indicating that presepsin is a useful rule-out test. The diagnostic odds ratio (DOR) was 12.31 (95% CI: 6.99–21.68; I² = 80.1%; Fig. 7). The area under the summary receiver operating characteristic (SROC) curve was 0.85 (Fig. 8), reflecting good diagnostic accuracy. The asymmetric SROC curve (Table 2), fitted using Moses’ model, produced an AUC of 0.8389 (SE = 0.0208) and a Q* of 0.7859 (SE = 0.0196), further supporting good diagnostic accuracy.

**Table 2:** Summary of pooled diagnostic accuracy estimates for presepsin

Summary Sensitivity Study | Sen [95% Conf. Iterval.] TP/(TP+FN) TN/(TN+FP)
Ruangsomboon2020 | 0.835 0.762 - 0.892 116/139 69/111 Piccioni2025 | 0.910 0.844 - 0.954 111/122 19/94 Jeong2022 | 0.703 0.598 - 0.795 64/91 104/169 Jereb2019 | 0.833 0.707 - 0.921 45/54 25/26 Ali2016 | 0.697 0.513 - 0.844 23/33 15/18 Lee2022 | 0.701 0.644 - 0.755 195/278 127/142 Yonaha2021 | 0.893 0.781 - 0.960 50/56 21/42 deGuadiana2017 | 0.671 0.549 - 0.779 47/70 105/130 Wang2020 | 0.818 0.673 - 0.918 36/44 88/98 Nakamura2019 | 0.809 0.667 - 0.909 38/47 327/393 Yamamoto2019 | 0.871 0.761 - 0.943 54/62 25/29
Pooled Sen | 0.782 0.755 - 0.807
Heterogeneity chi-squared = 44.72 (d.f.= 10) *p* = 0.000 Inconsistency (I-square) = 77.6% No. studies = 11. Filter OFF Add 1/2 to all cells of the studies with zero Summary Specificity Study | Spe [95% Conf. Iterval.] TP/(TP+FN) TN/(TN+FP)
Ruangsomboon2020 | 0.622 0.525 - 0.712 116/139 69/111 Piccioni2025 | 0.202 0.126 - 0.298 111/122 19/94 Jeong2022 | 0.615 0.538 - 0.689 64/91 104/169 Jereb2019 | 0.962 0.804 - 0.999 45/54 25/26 Ali2016 | 0.833 0.586 - 0.964 23/33 15/18 Lee2022 | 0.894 0.832 - 0.940 195/278 127/142 Yonaha2021 | 0.500 0.342 - 0.658 50/56 21/42 deGuadiana2017 | 0.808 0.729 - 0.872 47/70 105/130 Wang2020 | 0.898 0.820 - 0.950 36/44 88/98 Nakamura2019 | 0.832 0.791 - 0.868 38/47 327/393 Yamamoto2019 | 0.862 0.683 - 0.961 54/62 25/29
Pooled Spe | 0.739 0.714 - 0.763
Heterogeneity chi-squared = 221.35 (d.f.= 10) *p* = 0.000 Inconsistency (I-square) = 95.5% No. studies = 11. Filter OFF Add 1/2 to all cells of the studies with zero Summary Positive Likelihood Ratio (Random effects model) Study | LR+ [95% Conf. Iterval.] % Weight
Ruangsomboon2020 | 2.206 1.718 - 2.831 10.30 Piccioni2025 | 1.140 1.015 - 1.281 10.50 Jeong2022 | 1.829 1.449 - 2.308 10.33 Jereb2019 | 21.667 3.159 - 148.62 4.29 Ali2016 | 4.182 1.453 - 12.037 7.33
Lee2022 | 6.640 4.090 - 10.782 9.66 Yonaha2021 | 1.786 1.302 - 2.449 10.16 deGuadiana2017 | 3.491 2.367 - 5.149 9.96 Wang2020 | 8.018 4.385 - 14.663 9.23 Nakamura2019 | 4.814 3.711 - 6.246 10.28 Yamamoto2019 | 6.315 2.529 - 15.765 7.94
(REM) pooled LR+ | 3.530 2.102 - 5.926
Heterogeneity chi-squared = 279.75 (d.f.= 10) *p* = 0.000 Inconsistency (I-square) = 96.4% Estimate of between-study variance (Tau-squared) = 0.6623 No. studies = 11. Filter OFF Add 1/2 to all cells of the studies with zero Summary Negative Likelihood Ratio (Random effects model) Study | LR- [95% Conf. Iterval.] % Weight
Ruangsomboon2020 | 0.266 0.178 - 0.397 11.11 Piccioni2025 | 0.446 0.223 - 0.891 6.22 Jeong2022 | 0.482 0.344 - 0.676 12.54 Jereb2019 | 0.173 0.095 - 0.316 7.42 Ali2016 | 0.364 0.208 - 0.635 8.11 Lee2022 | 0.334 0.276 - 0.403 16.06 Yonaha2021 | 0.214 0.095 - 0.484 4.96 deGuadiana2017 | 0.407 0.288 - 0.575 12.37 Wang2020 | 0.202 0.108 - 0.380 7.01 Nakamura2019 | 0.230 0.128 - 0.415 7.61 Yamamoto2019 | 0.150 0.077 - 0.290 6.58
(REM) pooled LR- | 0.297 0.240 - 0.367
Heterogeneity chi-squared = 23.17 (d.f.= 10) *p* = 0.010 Inconsistency (I-square) = 56.8% Estimate of between-study variance (Tau-squared) = 0.0636 No. studies = 11. Filter OFF Add 1/2 to all cells of the studies with zero Summary Diagnostic Odds Ratio (Random effects model) Study | DOR [95% Conf. Iterval.] % Weight
Ruangsomboon2020 | 8.286 4.596 - 14.937 10.91 Piccioni2025 | 2.556 1.151 - 5.679 9.93 Jeong2022 | 3.793 2.196 - 6.549 11.10 Jereb2019 | 125.00 14.958 - 1044.6 4.51 Ali2016 | 11.500 2.711 - 48.777 6.85 Lee2022 | 19.892 10.987 - 36.013 10.90 Yonaha2021 | 8.333 2.944 - 23.592 8.73 deGuadiana2017 | 8.583 4.425 - 16.648 10.58 Wang2020 | 39.600 14.462 - 108.43 8.89 Nakamura2019 | 20.919 9.655 - 45.326 10.06 Yamamoto2019 | 42.188 11.607 - 153.33 7.53
(REM) pooled DOR | 12.310 6.990 - 21.678
Heterogeneity chi-squared = 50.16 (d.f.= 10) *p* = 0.000 Inconsistency (I-square) = 80.1% Estimate of between-study variance (Tau-squared) = 0.6733 No. studies = 11. Filter OFF Add 1/2 to all cells of the studies with zero Analysis of Diagnostic Threshold
Spearman correlation coefficient: 0.327 p-value= 0.326 (Logit(TPR) vs. Logit(FPR)
Moses’ model (D = a + bS)
Weighted regression (Inverse Variance) Var Coeff. Std. Error T p-value
a 2.601 0.233 11.142 0.0000 b(1) −0.442 0.153 2.887 0.0180
Tau-squared estimate = 0.3491 (Convergence is achieved after 5 iterations) Restricted Maximum Likelihood estimation (REML) No. studies = 11 Filter OFF Add 1/2 to all cells of the studies with zero
Meta-Regression(Inverse Variance weights) Var Coeff. Std. Err. p - value RDOR [95%CI]
Cte. 2.171 2.8113 0.4622 ---- ---- S −0.434 0.1788 0.0413 ---- ---- LogCutOff 0.072 0.4523 0.8781 1.07 (0.38;3.05)
Tau-squared estimate = 0.4176 (Convergence is achieved after 5 iterations) Restricted Maximum Likelihood estimation (REML) No. studies = 11 Filter OFF Add 1/2 to all cells of the studies with zero

### Heterogeneity and threshold effects

Considerable heterogeneity was detected, with I² values of 77.6% for sensitivity (χ² = 44.72, df = 10, *p* < 0.001) and 95.5% for specificity (χ² = 221.35, df = 10, *p* < 0.001). The between-study variance (τ²) estimated from the meta-regression model was 0.4176.

To explore whether the choice of cut-off value contributed to this heterogeneity, we assessed the threshold effect. Spearman’s correlation between the logit of the true-positive rate and the logit of the false-positive rate was 0.327 (*p* = 0.326), indicating no significant threshold effect. However, the Moses-Littenberg model (weighted regression of D against S) produced a slope of − 0.442 (*p* = 0.018), which would conventionally suggest a mild threshold effect. This discrepancy is not uncommon; Spearman’s correlation has lower power to detect non-linear threshold effects.

Importantly, meta-regression using Log(CutOff) as a covariate showed that the cut-off value did not significantly influence diagnostic accuracy (coefficient = 0.072, *p* = 0.878, RDOR = 1.07, 95% CI: 0.38–3.05; Table 3). Consequently, the observed heterogeneity is more likely attributable to clinical and methodological factors – such as study setting (ICU vs. non-ICU), geographical region (Asia vs. non-Asia), and study design – rather than the diagnostic threshold alone.

**Table 3:** Meta-Regression

Var	Coeff.	Std. Err.	*p* – value	RDOR	[95%CI]
Cte.	2.171	2.8113	0.4622	----	----
S	−0.434	0.1788	0.0413	----	----
LogCutOff	0.072	0.4523	0.8781	1.07	(0.38;3.05)

### Subgroup and sensitivity analyses

Due to the limited number of studies (*n* = 11), formal subgroup meta-analyses according to geographical region or clinical setting were not performed. Instead, meta-regression with Log(Cutoff) was conducted to explore the main source of heterogeneity (see Meta-regression above and Table 3).

A sensitivity analysis was performed excluding the Piccioni [16] study, which was identified as a statistical outlier because of its very low cut-off (165 pg/mL) and extremely low specificity (0.202). After exclusion, the pooled specificity increased from 0.739 (95% CI: 0.714–0.763) to 0.773 (95% CI: 0.696–0.835), and the pooled sensitivity increased from 0.782 (95% CI: 0.755–0.807) to 0.803 (95% CI: 0.739–0.854). Heterogeneity for specificity decreased from I² = 95.5% to 85.6%. Thus, this outlier study did affect the pooled estimates and contributed to the observed variability. The overall conclusion (presepsin as a useful rule-out test) remained unchanged.

### Publication bias

Deeks’ funnel plot asymmetry test was performed to assess publication bias. The test was not significant (*p* = 0.33, > 0.10), indicating no evidence of major publication bias among the 11 included studies.

## Discussion

This meta-analysis is the first systematic review and meta-analysis focusing exclusively on studies that adopted the Sepsis-3 criteria for diagnosing sepsis in adult patients (including both ICU and non-ICU settings). Our pooled estimates – sensitivity 78.2% (95% CI: 75.5–80.7%), specificity 73.9% (95% CI: 71.4–76.3%), and asymmetric SROC AUC of 0.84 – indicate good diagnostic accuracy for presepsin. The negative likelihood ratio (NLR) of 0.30 (95% CI: 0.24–0.37) confirms that presepsin is a useful rule-out test: a negative result reduces the post-test probability of sepsis to approximately one-third of the pre-test probability. In contrast, the positive likelihood ratio (PLR) of 3.5 (95% CI: 2.1–5.9) provides only a modest increase in the probability of disease when the test is positive. Therefore, presepsin is best suited for ruling out sepsis, particularly in settings with low to moderate pre-test probability (e.g., emergency departments), where rapid exclusion can avoid unnecessary antibiotic administration and invasive procedures [21, 25–28].

### Comparison with previous meta-analyses

Our sensitivity (78%) and specificity (74%) are slightly lower than those reported in earlier meta-analyses that mixed Sepsis-1/2/3 criteria (often > 80%). This difference is expected because Sepsis-3 defines a more severely ill population (requiring organ failure), which may alter test performance. The AUC of 0.84 is classified as good (not excellent), moderating previous overstatements [3, 10, 12]. A recent network meta-analysis of post-Sepsis-3 studies ranked presepsin third among sepsis biomarkers after CD64 and sTREM-1, with a fixed odds ratio of 10.04, and reported higher sensitivity than the qSOFA score [13]. Our findings align with that ranking and further emphasize the rule-out utility of presepsin.

### Heterogeneity and threshold effects

Considerable heterogeneity was observed (I² = 77.6% for sensitivity, 95.5% for specificity; τ² = 0.42). Due to the limited number of studies (*n* = 11), formal subgroup meta-analyses according to geographical region or clinical setting were not performed. Instead, meta-regression with Log(Cutoff) was conducted to explore the main source of heterogeneity (Table 3). The threshold effect assessment gave discrepant results: Spearman’s correlation was non-significant (*r* = 0.327, *p* = 0.326), but the Moses-Littenberg slope was significant (b = − 0.442, *p* = 0.018), indicating a mild threshold effect. This discrepancy is not uncommon; Spearman’s correlation has lower power to detect non-linear threshold effects. Importantly, meta-regression showed that the numerical cut-off value (log-transformed) did not significantly influence diagnostic accuracy (coefficient = 0.072, *p* = 0.878, RDOR = 1.07; Table 3). Therefore, the wide variation in cut-offs (165–1,315 pg/mL) explains only part of the heterogeneity; other clinical and methodological factors (e.g., disease severity, ICU vs. emergency department setting) may also contribute, but could not be formally tested due to the small number of studies.

### Outlier study and sensitivity analysis

The Piccioni study [16] was a statistical outlier because of its very low cut-off (165 pg/mL) and extremely low specificity (0.20). As reported in the sensitivity analysis, excluding this study increased pooled specificity from 0.739 to 0.773 and pooled sensitivity from 0.782 to 0.803, while reducing heterogeneity for specificity (I² from 95.5% to 85.6%). This outlier did affect the pooled estimates, but the overall conclusion (presepsin as a useful rule-out test) remained unchanged.

### Clinical implications and need for standardization

The wide range of reported cut-off values (165–1,315 pg/mL) and the lack of a universally accepted threshold currently limit clinical generalizability. Studies that demonstrated high accuracy used cut-offs mostly within 500–900 pg/mL [21, 22, 24]. We therefore recommend that future research focus on standardizing the presepsin cut-off, preferably through large, prospective, multicenter studies. Until then, presepsin should be used as an adjunct to clinical assessment (e.g., qSOFA) rather than as a standalone diagnostic test, particularly in resource-poor settings where rapid rule-out of sepsis can have a major impact [5, 29].

### Limitations

Several limitations must be acknowledged. First, the review was not registered in PROSPERO, which reduces transparency. Second, most included studies were single-center, limiting generalizability. Third, despite restricting to Sepsis-3 criteria, residual heterogeneity (τ² = 0.42) remained, indicating unmeasured clinical or methodological differences. Fourth, the wide range of cut-offs (165–1,315 pg/mL) and incomplete reporting of some thresholds hindered direct clinical translation. Fifth, individual patient data were not available for more sophisticated analyses (e.g., decision curve analysis). Finally, although Deeks’ test showed no publication bias (*p* = 0.33), selection bias cannot be excluded because of the predominance of single-center cohorts.

## Conclusions

This meta-analysis is the first focused analysis restricted exclusively to studies that used Sepsis-3 criteria and plasma presepsin measurement for the diagnosis of sepsis in adult patients (including both ICU and non-ICU settings). Our findings indicate that presepsin has good diagnostic accuracy (AUC 0.84, pooled sensitivity 78%, specificity 74%). The negative likelihood ratio of 0.30 confirms that presepsin is a useful rule-out test, capable of reducing the post-test probability of sepsis to approximately one-third when the test is negative. This performance characteristic supports its potential role as a decision-support instrument, particularly in emergency settings where rapid exclusion of sepsis can guide clinical management. However, the wide variation in reported cut-off values (165–1,315 pg/mL) and the lack of a universally accepted threshold currently limit clinical generalizability. Therefore, while presepsin shows promise as an adjunctive biomarker for ruling out sepsis, standardization of cut-off values through large, prospective, multicenter studies is essential before it can be recommended for routine clinical practice.

## Data Availability

Data and material would be access by request to the corresponding authors.

## Abbreviations

AKI: Acute Kidney Injury
AUC: Area Under the Curve
CI: Confidence Interval
CLEIA: Chemiluminescent Enzyme Immunoassay
CRP: C-Reactive Protein
DOR: Diagnostic Odds Ratio
ED: Emergency Department
ELISA: Enzyme-Linked Immunosorbent Assay
FN: False Negative
FP: False Positive
ICU: Intensive Care Unit
MeSH: Medical Subject Headings
NLR: Negative Likelihood Ratio
PCR: Polymerase Chain Reaction
PICOS: Population, Intervention, Comparator, Outcome, Study design
PLR: Positive Likelihood Ratio
PRISMA-DTA: Preferred Reporting Items for Systematic Reviews and Meta-Analyses of Diagnostic Test Accuracy Studies
qSOFA: Quick Sequential Organ Failure Assessment
QUADAS-2: Quality Assessment of Diagnostic Accuracy Studies-2
REML: Restricted Maximum Likelihood
SIRS: Systemic Inflammatory Response Syndrome
SOFA: Sequential Organ Failure Assessment
SROC: Summary Receiver Operating Characteristic
sTREM-1: Soluble Triggering Receptor Expressed on Myeloid cells-1
TP: True Positive
TN: True Negative
